# Changes in the Content of Dietary Fiber, Flavonoids, and Phenolic Acids in the Morphological Parts of *Fagopyrum tataricum* (L.) Gaertn Under Drought Stress

**DOI:** 10.3390/molecules30020270

**Published:** 2025-01-11

**Authors:** Krzysztof Dziedzic, Pathumi Ariyarathna, Artur Szwengiel, Marzanna Hęś, Karolina Ratajczak, Danuta Górecka, Hanna Sulewska, Jarosław Walkowiak

**Affiliations:** 1 Department of Food Technology of Plant Origin, Poznan University of Life Sciences, Wojska Polskiego 31, 60-624 Poznan, Poland; 2 Department of Pediatric Gastroenterology and Metabolic Diseases, Poznan University of Medical Sciences, Szpitalna 27/33, 60-572 Poznan, Poland; jarwalk@ump.edu.pl; 3 Department of Agriculture, Sri Lanka School of Agriculture, Dambulla 21100, Sri Lanka; pathumi1991@gmail.com; 4 Department of Gastronomic Technology and Functional Foods, Poznan University of Life Sciences, Wojska Polskiego 31, 60-624 Poznan, Poland; marzanna.hes@up.poznan.pl (M.H.); danuta.gorecka@up.poznan.pl (D.G.); 5 Department of Agronomy, Poznan University of Life Sciences, Dojazd 11, 60-632 Poznan, Poland; karolina.ratajczak@up.poznan.pl (K.R.); hanna@sulewski.pl (H.S.)

**Keywords:** tartary buckwheat, antioxidative substances, abiotic stress, leaves, stalk, seed, husk

## Abstract

Background: Tartary buckwheat is a plant recognized for its resistance to various environmental stresses. Due to its valuable source of phenolic compounds, *Fagopyrum tataricum* is also characterized as a medicinal plant; therefore, the aim of this study was to investigate the drought stress for the levels of phenolic compounds in the morphological parts of the plant. Methods: This experiment was conducted in 7 L pots under laboratory conditions. Phenolic compounds were identified using a UHPLC–MS chromatography system. Antioxidant activity was assessed using well-known methods, including the DPPH scavenging activity and ferrous ion chelating activity. Results: In Tartary buckwheat leaves, stems, seeds, and husks, 57 phenolic compounds were identified, with a predominance of quercetin 3-rutinoside, quercetin, kaempferol-3-rutinoside, kaempferol, and derivatives of coumaric acid. It was observed that the Tartary buckwheat samples subjected to drought stress exhibited a slight decrease in the majority of individual phenolic compounds. Conclusions: The measurement of biological parameters indicated that plant regeneration after drought stress demonstrated a rapid recovery, which can be a positive response to the progression of climate changes.

## 1. Introduction

Many species of buckwheat are found worldwide; however, only two are recognized for their agricultural and nutritional significance [1]. These two species, Common buckwheat (*Fagopyrum esculentum* Moench) and Tartary buckwheat (*Fagopyrum tataricum* L. Gaertn), are widely utilized as raw material for food production. These grains are renowned for their rich nutritional profile, containing low digestible polysaccharides such as dietary fiber. Additionally, buckwheat grains provide proteins with well-balanced amino acids, lipids, essential micronutrients like minerals and vitamins, and polyphenolic compounds, including flavonoids and phenols with antioxidative properties [1,2,3].

The major components of dietary fiber are non-starch polysaccharides and lignin, which are concentrated in the cell walls of starchy endosperm, aleurone, seed coats, and hulls [4]. A considerable portion of buckwheat dietary fiber consists of soluble fraction. The soluble non-starch polysaccharides of buckwheat are rich in xylose, mannose, galactose, and glucuronic acid [5]. Buckwheat grains and by-products are well known for their significant amounts of dietary fiber content and antioxidative substances. The literature provides considerable evidence of an additive relationship between insoluble dietary fiber fractions, especially lignin, and total phenolic acid content [6,7,8]. Lignin contains numerous phenolic hydroxyl groups and is an abundant source of polyphenolic compounds that can terminate oxygen reaction chains and neutralize harmful free radicals [9]. Regarding pro-health food products, buckwheat can be used as an effective raw material for their production. Buckwheat and its products exhibit significant antioxidative activity, contributing to their prophylactic properties, which are crucial for human health [1]. It contains catechins and phenolic acids, including hydroxybenzoic, synegric, *p*-hydroxy-benzoic, vanillic, and *p*-coumaric acids, which exhibit strong antioxidant properties. These compounds are particularly concentrated in the bran and aleurone layer of buckwheat grains. However, the primary antioxidants in buckwheat are rutin, quercetin, and catechin [1,3,10]. Notably, buckwheat bran and hulls demonstrate two to seven times higher antioxidant activity compared to barley, triticale, and oats. The rutin content in buckwheat varies based on genotype, growing conditions, developmental phase, plant part, and year of harvest [11]. Epidemiological studies have suggested that dietary flavonoids play a crucial role in the prevention of coronary heart diseases and cancers [12]. Moreover, products rich in dietary fiber are associated with the prevention of affluent diseases such as overweight, obesity, type 2 diabetes, cardiovascular disease, and colon cancer. As a result of these health benefits, the popularity of common buckwheat products has gained increasing attention in recent years, especially among health-conscious consumers. 

It is worth noting that interest in Tartary buckwheat is increasing in some Asian and European countries. Tartary buckwheat deserves special attention because it is often underestimated and even treated by farmers as a weed, resulting in its cultivation being relatively uncommon. Compared to Common buckwheat, Tartary buckwheat contains a significant amount of rutin, quercetin, dietary fiber, vitamins, minerals, and essential amino acids. Due to these attributes, Tartary buckwheat is recognized as a medicinal plant and has significant potential to be utilized in the production of dietary supplements. Furthermore, Tartary buckwheat may play an important role in reducing the risk of developing insulin resistance [13,14]. Its grains contain protein-derived peptides with superior antioxidant capacity. Among these, four peptides (CR-8, LR-8, GK-10, and SR-12) have been identified for their ability to mitigate mild damage caused by reactive oxygen species [15]. Moreover, polyphenols extracted from Tartary buckwheat husk through numerous methods have exhibited significant cytotoxicity against HepG2 cancer cells [16]. 

Tartary buckwheat, compared to Common buckwheat, is characterized by higher yield potential (around 1.5 ton/ha), superior frost resistance, and the ability to grow at higher elevations [1,14]. Due to its low agrotechnical requirements and health-promoting properties, Tartary buckwheat is gaining increasing attention from researchers, food producers, and farmers. This provides hope for new possibilities in food technology and human nutrition, especially in the context of global climate change. Buckwheat flavonoids, as a secondary metabolite, play a significant role in regulating plant’s response to abiotic environmental factors. Consequently, many researchers are focusing on the stress adaptability of plants, aiming to understand the relationship between environmental stress adaptation and secondary metabolite production [17]. Buckwheat tissues can be a rich source of high-quality flavonoids; however, their content varies throughout the plant development and is significantly influenced by soil quality [18]. 

Both dietary fiber and flavonoids are important bioactive components that play a key role in protecting against chronic diseases and prolonging the shelf life of food products. Certain morphological parts of Tartary buckwheat, which are rich in these substances, can be utilized to create healthy food products, particularly in the context of climate change. Drought, a consequence of climate change, severely limits agricultural development, which leads to a reduction in crop yield and quality [19,20]. Buckwheat is considered tolerant or even resistant to adverse environmental and abiotic stresses, including drought [21]. The regulatory mechanism of Tartary buckwheat under drought stress is still under investigation [22]. In databases, there are many studies describing the mechanisms of internal protection systems against drought stress in Tartary buckwheat [23,24,25,26,27]. The authors of these studies pay special attention to the expression of antioxidative enzymes, such as peroxidase (POD), superoxide dismutase (SOD), catalase (CAT), and others, and also identify the concentrations and activities of non-enzymatic total phenolic compounds. It is well known that individual phenolic substances can exhibit different antioxidant activities; therefore, it is important to determine their profiles and concentrations.

Considering the lack of evidence describing the effect of abiotic stress—among others, drought—on the content of individual phenolic compounds, which have been previously described as health-promoting factors, it seems appropriate to estimate selected pro-health compounds (individual flavonoids and phenolic acids). Based on previous studies, we hypothesize that drought stress in Tartary buckwheat significantly affects the content of 57 individual phenolic compounds. Tartary buckwheat, recognized as a medicinal plant, is a rich source of these compounds. Therefore, both the green parts of the plant (for the pharmaceutical industry) and the grains (for the food industry) were considered in this study.

## 2. Results

### 2.1. Chemical Characteristics of the Samples

Table 1 presents the chemical characteristics of well-watered (N) and drought-stressed (S) morphological parts of Tartary buckwheat. All samples were obtained as dried and not fragmented morphological parts of the plant. The dry matter content varied from 88.91% in the non-stressed seeds to 93.43% in the stressed stem samples. No significant differences were observed in moisture content between the drought-stressed and well-watered samples. Regarding the abundance of the compounds investigated, proteins were the second most abundant. The highest protein content was observed in the non-stressed seeds and leaves (16.11% and 12.89%, respectively). The water conditions significantly affected the protein content in almost all samples; however, no changes in protein levels were observed in the husk, likely due to its low total protein content, which ranged from around 8.23% to 8.59%. Limited water availability reduced total protein content by approximately 24%, 26%, and 44% in leaves, seeds, and stems, respectively. Tartary buckwheat samples also had low lipid content. The highest lipid level was observed in non-stressed seeds (2.58%), while the lowest was found in both stressed and non-stressed stems (approximately 0.47–0.57%). The results indicate that different water conditions did not significantly affect the total lipid content in stems, seeds, and husks. However, a statistically significant difference in lipid content was observed in leaves, with values of 2.44% for non-stressed leaves and 1.74% for stressed leaves. Total dietary fiber (TDF), particularly its insoluble fraction (IDF), was abundant across all morphological parts of Tartary buckwheat. The highest TDF levels were detected in both variants of the stems (76.20–76.65%), while seeds contained the lowest levels (approximately 15.5%). In contrast to the other samples, only the husk samples showed a significant increase in the TDF content with water availability, approximately 16%. Similarly, in the IDF fraction, higher values were identified in stems, with 72.16% and 73.19% for the non-stressed (N) and stressed (S) variants, respectively. Other morphological parts of the plant exhibited similar TDF patterns, with no significant differences based on water supply. The soluble dietary fiber (SDF) content ranged from 0.03% to 0.09% in non-stressed and stressed seeds, respectively. In non-stressed stem samples, SDF levels were notably higher, reaching 4.04%. Water conditions significantly affected the SDF fraction in all analyzed samples except for seeds. It is important to emphasize that despite the low SDF content, this fraction plays a crucial role in human well-being. For this reason, the proportion of SDF to IDF deserves special attention. This proportion varied depending on both the plant’s morphological parts and the water variant. The highest SDF to IDF deserves special attention. This proportion was found to vary depending on both the plant’s morphological parts and the water variant. The highest SDF-to-IDF ratio was observed in stems and husk samples, ranging from 1:18 to 1:26, in contrast to leaves and seeds, which showed much lower ratios of 1:114 to 1:519, respectively. The water treatment method had a significant effect on the SDF/IDF ratio in both stem and seed samples. In stems, well-watered conditions shifted the ratio from 1:21 (SDF/IDF) to 1:18 (SDF/IDF). In seeds, however, an opposite trend was observed, with water treatment altering the SDF/IDF ratio from 1:170 to 1:519.

### 2.2. Total Phenolic Compounds and Antioxidative Activity

Table 2 presents DPPH scavenging activity, ferrous ion chelating activity, total phenolic content (TPC), and antioxidative compounds measured through HPLC–MS. The antioxidative potential was assessed through DPPH scavenging activity. The highest DPPH antioxidative capacities were observed in non-stressed leaves (257.2 mg Trolox/g dry matter of extract) and stressed leaves (208.0 mg Trolox/g dry matter of extract), compared to other samples. In the pot experiment, water treatment significantly increased the DPPH scavenging capacity in leaf and seed samples by approximately 24% and 22%, respectively. No significant increase was identified in stems or seeds. In contrast, the highest ferrous ion chelating activity was recorded in the non-stressed husk (439.4 mg EDTA/g dry matter of extract) and stressed husk (210.5 mg EDTA/g dry matter of extract), while the lowest activity was recorded in non-stressed and stressed leaves (2.2–3.5 mg EDTA/g dry matter of extract, respectively). Measurements of ferrous ion chelating activity showed that water conditions did not affect leaves and stems. However, a notable effect was observed in seeds, with values of 57.9 mg EDTA/g dry matter for stressed seeds and 81.2 mg EDTA/g dry matter for non-stressed seeds. In the husk, the effect was particularly pronounced, with an increase of approximately 208.8%. This study identified some discrepancies between the results obtained from HPLC–MS and TPC measurements. The highest TPC levels were observed in the stressed and non-stressed leaves (296.18 and 152.76 mg/g, respectively), while the lowest was in stressed and non-stressed seeds (28.94 and 31.19 mg/g, respectively). Water treatment significantly influenced all morphological parts of Tartary buckwheat, except for the husk (the outer layer of seeds), where no meaningful effect was observed. Notably, TPC levels in leaves were higher under limited water conditions (296.18 mg/g dry matter). In contrast, stems and seeds cultivated under well-watered conditions exhibited higher TPC levels, approximately 12% and 8%, respectively. According to HPLC–MS results, the highest levels of antioxidative substances, expressed as the sum of total identified compounds, were found in non-stressed leaves and husks (309.3 and 276.8 mg/100 g dry matter, respectively). In all samples, water conditions significantly influenced the total amount of these compounds. Drought stress led to a decrease in these compounds across all analyzed samples, with seeds and leaves showing decreases of approximately 5% and 14%, respectively, while stems and husks exhibited decreases of about 10%. 

### 2.3. Individual Antioxidative Compounds (HPLC–MS)

**Figure 1:**
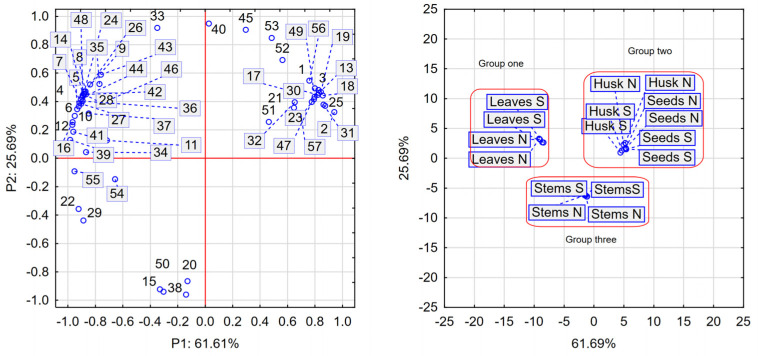
Two-dimensional plots representing the PCA: loading plot (left site) and score plot (right site). 1—Dihydroxybenzoate hexoside; 2—Dihydroxybenzoate hexoside; 3—Vanillic acid glucoside; 4—Dihydroxybenzoate hexoside; 5—Neochlorogenic acid; 6—Dihydroxybenzoate; 7—Catechin glucoside; 8—Catechin 3-*O*-rutinoside; 9—Ferulic acid; 10—Hydroxycinnamic acid; 11—Chlorogenic acid; 12—Catechin; 13—Quercetin glycoside; 14—Procyanidin B2/B4; 15—Ethyl syringate; 16—Sinapoyl hexoside; 17—Coumaroylquinic acid; 18—Epiafzelechin–catechin; 19—Kaempferol glucoside; 20—Apigenin glycoside; 21—Coumaroylquinic acid; 22—*p*-Coumaric acid; 23—Hydroxycoumarin; 24—Myricetin 3-glucoside; 25—Afzelechin; 26—Flavonoid glycoside; 27—Chrysoeriol glucoside; 28—Flavonoid glycoside; 29—Ferulic acid; 30—Quercetin 3-rhamninoside; 31—Myricetin; 32—Quercetin glycoside; 33—Luteolin–glucoside; 34—Epicatechin/catechin gallate; 35—Quercetin 3-rutinoside; 36—Procyanidin B; 37—Quercetin hexoside; 38—Sinapoyl aldehyde; 39—Coumaric acids derivative; 40—Kaempferol-3-rutinoside; 41—Isorhamnetin glycoside; 42—Quercetin glycoside; 43—Quercetin glycoside; 44—Phloretin glucoside; 45—Quercetin glycoside; 46—Emodin glycoside; 47—Epicatechin hydroxybenzoate; 48—Flavonoid glycoside (Luteolin glycoside); 49—Flavonoid glycoside (Kaempferide 3-rhamnoside-7-(6″″-succinylglucoside)); 50—Feruloyltyramine; 51—Malvidin glucoside–ethyl–catechin; 52—Flavone (Myricetin); 53—Quercetin; 54—Luteolin; 55—Methylquercetin; 56—Kampherol; 57—Dimethylquercetin.

A total of 57 compounds were identified in the analyzed samples. In the supplementary results table, some records appear multiple times as CSI:Finger ID identified several similar structures with high scores. For example, it lists dihydroxybenzoate hexoside 1, 2, and 3; ferulic acid 1 and 2; and quercetin glycoside 1, 2, 3, 4, and 5 (Table S1). The quantities of the presented compounds varied widely. Minimum values were found for hydroxycinnamic acid (≤1.6 µg/g), hydroxycoumarin (≤0.67 µg/g), afzelechin (≤0.44 µg/g), ferulic acid (≤0.76 µg/g), quercetin 3-rhamninoside (≤0.18 µg/g), and feruloyltyramine (≤2.86 µg/g). In contrast, the highest levels were identified for quercetin 3-rutinoside (706–1374 µg/g), quercetin (95–725 µg/g), kaempferol-3-rutinoside (125–484 µg/g), campherol (5–350 µg/g), and coumaric acids derivatives (4–121 µg/g). The content of the main phenolic compounds varied among the investigated morphological parts of the plant. Figure 1 provides a statistical presentation of individual phenolic compounds found in the leaves (S and N), stalks, seeds, and husks. No significant impact of water supply on the levels of the 57 identified phenolic compounds was observed. According to this observation, only the morphological parts of the plant and the separated husk formed three distinct groups: the first group included leaves (N and S); the second group comprised husk and seeds (N and S); and the third group contained stems (N and S). The most abundant phenolic compound found in the leaves was quercetin 3-rutinoside (rutin), with concentrations of 1374 µg/g in leaves (N) and 1121 µg/g in leaves (S), while seeds had the lowest levels (693 µg/g in N seeds and 679 µg/g in S seeds). The husk and seeds were dominated by compounds such as dihydroxybenzoate hexoside (1), vanillic acid glucoside (3), quercetin glycoside (13 and 32), epiafzelechin–catechin (18), kaempferol glucoside (19), coumaroylquinic acid (21), hydroxycoumarin (23), afzelechin (25), quercetin 3-rhamnoside (30), epicatechin hydroxybenzoate (47), and kaempferol (57). Notably, epiafzelechin–catechin, hydroxycoumarin, and quercetin 3-rhamnoside, although present in trace amounts, were found exclusively in the husk and seeds. The final group comprising stems contained ethyl syringate (15), apigenin glycoside (20), sinapoyl aldehyde (38), and feruloyltyramine (50).

### 2.4. Physiological Plant Parameters Under Water Drought Stress

**Figure 2:**
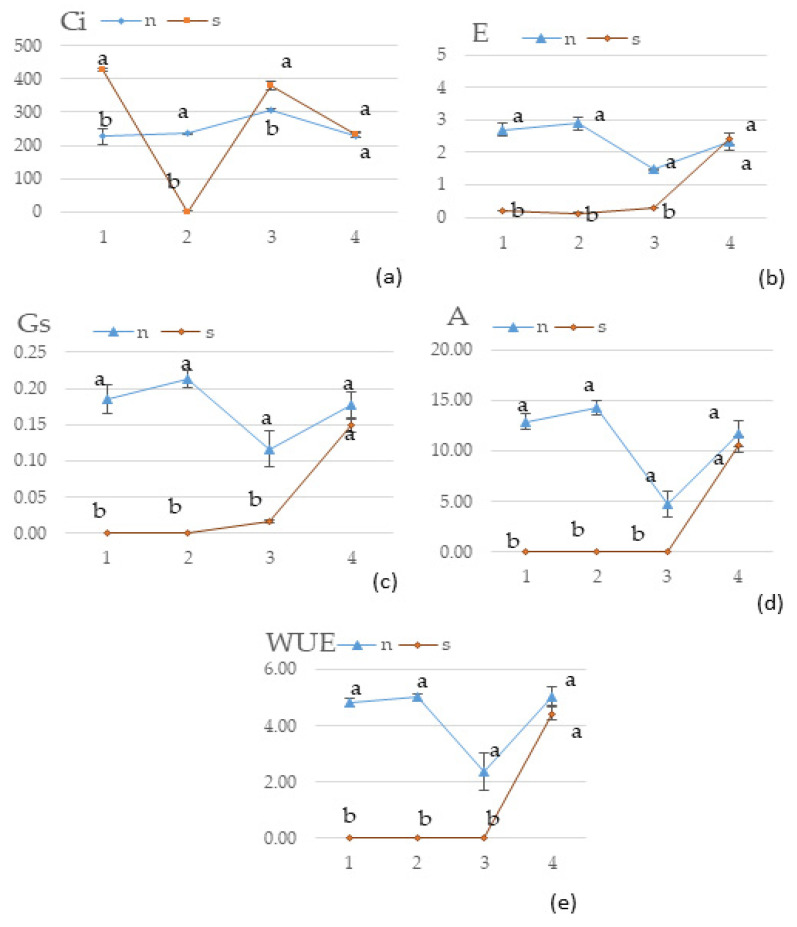
Effect of water stress on (**a**) the intercellular CO_2_ concentration (µmol∙m^−1^) (Ci), (**b**) the transpiration rate (mmol∙m^−2^∙s^−1^) (E), (**c**) the leaf stomatal conductance (mol∙m^−2^∙s^−1^) (Gs), (**d**) the net photosynthesis rate (mmol∙m^−2^∙s^−1^) (A), (**e**) and the water use efficiency (WUE) of fresh plant *F. tataricum*. Water treatment: N—non-stressed; S—stressed; 1, 2—days of measurements after the occurrence of stress; 3, 4—days of measurement after regeneration; the vertical bars show standard errors across treatment means; ^a–b^ different letters mean a significant difference (*p* < 0.05) between the two water treatments according to ANOVA test.

Figure 2 illustrates the physiological state of plants during drought stress. A significant impact of water stress was observed on all tested parameters, resulting in a decrease in the values of most parameters, except for the intercellular CO_2_ concentration (Ci). The Ci value in S was 50% higher than in N; however, by the second time of measurement (18th day of occurrence of stress), the Ci level dropped drastically. The effect of plant regeneration was confirmed, as all tested parameters showed increased values to the extent that no significant differences were demonstrated between S and N.

Based on the analysis of the chlorophyll fluorescence measurement results (Table 3), no significant effect of stress was observed. There were no significant differences between the maximum quantum efficiency of PSII photochemistry (Fv/Fm) and the effective quantum yield of photosystem II (Y). However, during drought stress, the level of electron transport rate (ETR) was 29.7 higher in S compared to N. After regeneration, there were no significant differences in all the tested chlorophyll fluorescence parameters between S and N, suggesting a complete recovery of the plants to a healthy state.

## 3. Discussion

Experiments comparing the morphological parts of drought-stressed and non-stressed Tartary buckwheat plants revealed no significant differences in moisture content across all analyzed samples. However, other studies have reported that moisture content varied depending on the duration of drought stress treatments and the plant varieties used in the experiments. These studies highlighted that different plant varieties exhibited markedly distinct results under short- and long-term drought stress treatments [28]. Therefore, the selection of more drought-resistant Tartary buckwheat plants for cultivation appears to be highly important. Similarly, a previous study investigated Amaranthus leaves under short, medium, and long drought stress treatments and observed a reduction in moisture content across all trials [29]. 

Buckwheat grains are generally a good source of protein, with a well-balanced amino acid composition [30]. Tartary buckwheat is typically characterized by a lower protein content compared to common buckwheat [30,31,32]. In our study, the total protein content was highest in dehulled seed samples, ranging between 11% and 16.1%, which is comparable to data reported in the literature (11–18.9%) [31,32]. The drought-stressed treatment altered the concentration of total proteins in grain samples, as shown in previous studies [30]. However, grains from well-watered plants exhibited higher protein concentrations. The second highest protein content was observed in the S and N husk samples, which contrasts with findings from other studies [31]. The protein content of approximately 8.5% in the husk fraction can be attributed to the dehulling process conducted under laboratory conditions. It is likely that parts of the grain’s outer layer, which is rich in protein, were crushed and transferred to the husk samples. No significant differences in protein levels were observed between the well-watered and drought-treated husk samples. Additionally, we identified slightly higher protein levels in leaves and stems compared to values reported by other studies. Yuan et al. (2024) detected predominantly enzymatic proteins in the green parts of Tartary buckwheat during growth, which are involved in the physiological functions of the plant [28]. Well-watered conditions led to an increase in the total protein content in leaves and stems. Other researchers have reported varying effects of well-watered and drought conditions on changes in soluble protein levels attributed to differences in plant varieties and the duration of drought treatment. Prolonged stress occasionally causes a significant decrease in soluble protein content [28]. A similar trend was observed in wheat plants treated with drought stress, as demonstrated in our study [33,34]. These studies reported a significant decrease in soluble proteins in the green parts of wheat. Additionally, drought stress at different stages of plant growth has been reported to impact the regulation of carbon and nitrogen metabolism [35], which may explain the changes in protein content depending on water conditions. 

*Fagopyrum tataricum* is not considered an oilseed plant due to its relatively low lipid content. However, its fats are rich in tocopherols and phytosterols [36]. The highest concentration of buckwheat lipids is typically found in its seeds and bran. In this experiment, variations in total lipid content were observed depending on the environmental treatment; however, these changes were significant only in the leaf samples. Yin et al. (2024) reported that water limitation during corn plant growth did not significantly affect the total lipid content in its green tissues after regeneration, which contrasts with the findings of our study [37]. 

The morphological parts of Tartary buckwheat are a rich source of total dietary fiber, particularly its insoluble fraction [6,31,38]. All of our samples were characterized by a high content of total dietary fiber, with the insoluble dietary fiber (IDF) fraction being the dominant component, while the soluble dietary fiber (SDF) fraction was the smallest. The order of TDF contents, from highest to lowest, is as follows: stems > leaves > husk > seeds. A previous study has reported lower fiber concentrations in the well-watered morphological parts of the plant, as determined using the chemical method outlined by Van Soest [6]. There is limited information in the literature regarding the effect of drought treatment on TDF amounts and its distribution in the morphological parts of Tartary buckwheat. However, some studies have observed that drought stress did not alter the TDF content in wheat [34]. In contrast, another study has found that drought treatment of the Amaranthus plant led to an increase in total dietary fiber content [29]. Tartary buckwheat samples also contain the SDF fraction; however, the morphological parts of the plant are not considered rich sources of these compounds. The SDF fraction plays a vital role in enhancing gastrointestinal health, as it is linked to an increase in the microbiome and the production of various beneficial metabolites, such as short-chain fatty acids [4]. To increase the SDF fraction at the expense of the IDF fraction, mechanical fragmentation is recommended [39]. 

Tartary buckwheat is recognized as a medicinal plant due to its high antioxidant profile, up to 10 times higher than that of other cereals and pseudo-cereals [31]. As a result, leaves are commonly used in Western Asian countries to prepare Tartary buckwheat tea [4]. Several studies have reported that drought treatment caused stress during the growth of buckwheat plants, which is ultimately linked to the expression of genes responsible for increasing protective factors [28,35]. There is also some evidence suggesting that the cultivar and growing season influence the content of phenolic acids [31]. The antioxidant potential of Tartary buckwheat is strongly associated with its phenolic compound content [30]. Our results suggest that drought treatment decreased DPPH scavenging activity in the leaves and husks, although the leaves exhibited the highest total phenolic content. A similar trend for TPC was observed in both hull and leaves, contrary to findings from another study [28]. However, no significant differences were noted for dehulled grains. Moreover, well-watered dehulled seeds and husks exhibited higher ferrous ion chelating activity, partially contradicting previously conducted studies. A comparison of TPC and phenolic profile estimated by HPLC–MS revealed varied tendencies between drought and well-watered treatment samples, suggesting a lack of stress symptoms in the morphological parts of the treated plants. Drought treatment induces the reactive oxygen and nitrogen species (ROS and RNS), which regulate the state of the cell. The antioxidant system in plants is divided into two parts: enzymatic (catalase, superoxide dismutase, glutathione peroxidase, and others) and non-enzymatic (phenolic compounds). During stress conditions, the detoxification of ROS and RNS is carried out by an antioxidant system, as some glycosides and phenolic substances act as ROS scavengers; as a result, the concentration of phenolic compounds and their activity were lower in drought-treated samples [40,41]. It was notably observed that water deficit led to a decrease in most identified antioxidative compounds. Among these, phenolics such as rutin, quercetin, quercetin glucosides, and coumaric acid were found to be dominant. Both common and Tartary buckwheat are rich in these compounds. However, not all 57 compounds were identified in every sample, as their distribution varies during plant growth [30]. Principal component analysis (Figure 1) revealed that individual phenolic compounds grouped the samples into three clusters: leaves (group one), seeds and husks (group two), and stems (group three). Furthermore, no distinct group was generated by the drought-treated samples, providing strong evidence that Tartary buckwheat is a plant with good tolerance to water deficit. Plants respond to water-deficit conditions through a series of physiological, cellular, and molecular processes that enhance their stress tolerance [42]. Similar to our findings, some studies revealed that Tartary buckwheat exhibits physiological responses to drought stress, including a reduction in photosynthesis, transpiration, stomatal conductance, and yield with the increase in intercellular CO2 concentration [43]. Additionally, previous research showed that wheat genotypes with higher antioxidant activity increased intercellular CO_2_ concentration, improved water use efficiencies and net photosynthesis, and were better able to tolerate drought stress conditions [44].

Tartary buckwheat, as a medicinal plant, is an excellent source of various phenolic compounds with high biological activity. In addition to its significant total dietary fiber content, particularly its insoluble fraction, it contains 57 distinct phenolic compounds that are distributed across different morphological parts of the plant. This composition contributes effectively to the antioxidative activity of all parts of the plant. Furthermore, drought stress did not cause negative effects on the plant’s physiological development during its final growth stages. Therefore, Tartary buckwheat can be recommended as a drought-resistant plant, making it suitable for cultivation in the context of progressive climate changes. In this study, we identified the activity of phenolic compounds against oxygen and nitrogen-reactive species, which effectively protected the plant during the final stage of growth. For this reason, to obtain higher amounts of phenolic compounds and greater antioxidant activity, we recommend harvesting the plant during the early stage of drought treatment.

## 4. Materials and Methods

### 4.1. Material

Seeds of Tartary buckwheat (*Fagopyrum tataricum* Gaertn.) were obtained from the Breeding Station (Palikije, Poland) and were previously cultivated commercially. The experiment was conducted in 2022. They were sown in 7 L polyethylene pots filled with soil classified as luvisol, a light clay sand grade, shallowly deposited on light clay that belongs to a good rye complex. The soil had a pH of 5.4, with an average content of 120.6 mg P_2_O_5_ per kg of soil (very high), 122.5 mg K_2_O per kg of soil (high), and 0.59% organic matter. Five seeds were sown in each pot, and after germination, three uniformed plants were retained. The pot study was conducted in the didactic plant collection facility belonging to the Department of Agronomy at the Poznan University of Life Sciences. Four weeks after seed sowing, the plants were transferred to a phytotron (with a temperature of 22 ± 2 °C, relative humidity of 65 ± 5%, and a 16-h photoperiod). Half of the experimental pots (120 plants) were maintained under control conditions with water supplied every 3 days, while the other 120 plants were subjected to water stress, with no water supply for 2 weeks during the 69–73 BBCH phase (phenological development stages of plants). Soil moisture was measured to confirm well-watered conditions (15% water content, equating to 70% field capacity) or water stress conditions (<10% water content, approximately <40% field capacity) using a probe (ThetaProbe, Eijkelkamp Penetrologger SN, Giesbeek, The Netherlands). The experiment lasted 80 days. Samples of stalks, leaves, husks, and seeds were collected after harvesting and stored under chilled conditions (around 7 °C) for no longer than 6 months. All samples were ground using a knife mill (WŻ1, Sadkiewicz, Poland), ensuring that the size of each particle was no larger than 200 μm. A representative sample was taken in triplicate for each analysis.

### 4.2. Reagents

Caffeic acid (C0625-25G), chlorogenic acid (PHR2202-100MG), *p*-coumaric acid (C9008-1G), sinapic acid (D7927-5G), quercetin (Q4951-10G), 6-hydroxy-2,5,7,8-tetramethylchromane-2-carboxylic acid (Trolox, 238813-5G), and 2,2-diphenyl-1-picrylhydrazyl (DPPH, D9132-1G) were purchased from Sigma-Aldrich company (Darmstadt, Germany). The total dietary fiber enzymatic kit was obtained from Megazyme company (K-TDFR 05/12, Wicklow, Ireland). Ethylenediaminetetraacetic acid (EDTA, PA-03-4011-E#100G) was obtained from Pol-Aura company (Poznan, Poland). Methanol (67-56-1), diethyl ether (60-29-7), sulphuric acid VI (7664-93-9), sodium hydroxide (1310-73-2), and Kjeldahl Catalyst (85539, K_2_SO_4_, 23,10%; Na_2_SO_4_, 69,30%; CuSO_4_, 1,80%; TiO_2_, 2,80%) were purchased from VWR company (Gdansk, Poland). 

### 4.3. Chemical Composition

Total nitrogen compounds were estimated using the ISO Method 20483:2013 [45]. The results were calculated as protein content with a conversion factor of 6.25. The enzymatic method AOAC 991.43 was used to estimate total dietary fiber (TDF) and its soluble (SDF) and insoluble (IDF) fractions [46]. The AACC Method 30-10.01 was conducted to analyze total lipid content using the Soxtec System HT6 (Foss Tecator, Sweden) [47]. 

### 4.4. Selected Phenolic Substances- HPLC–MS Chromatography

Reversed-phase (C18 column) electrospray ionization mass spectrometry (RP-UHPLC-ESI-MS) analysis was performed using a Dionex UltiMate 3000 UHPLC (Thermo Fisher Scientific, Sunnyvale, CA, USA) coupled to a Bruker maXis impact ultrahigh resolution orthogonal quadrupole-time-of-flight accelerator (qTOF) equipped with an ESI source and operated in both positive- and negative-ion modes (Bruker Daltonik, Germany). The RP chromatographic separation was achieved with a Kinetex™ 1.7 µm C18 100 Å, LC column 100 × 2.1 mm (Phenomenex, Torrance, CA, USA) at 30 °C using 8 mM formic acid as eluent A and acetonitrile as eluent B with flow 0.2 ml/min. Molecular ions [M + H]^+^ and [M − H]^−^ for phenolic compounds were extracted from full scan chromatograms (±0.005 *m*/*z*), and peak areas were integrated using TASQ 2.1 (Bruker Daltonik, Bremen, Germany). The limit of quantification (LOQ where S/N > 15) was determined for caffeic acid, chlorogenic acid, *p*-coumaric acid, sinapic acid, and quercetin, and it was no lower than 0.01 µg/mL [48]. Calibration and quality control (QC) samples were prepared in a water–methanol mixture (30:70 *v*/*v*) as a surrogate matrix. The recovery of standards spiked into samples was above 95–106%. The coefficient of determination (R^2^) for all calibration curves was higher than 0.99. The compounds present in each sample were identified based on the retention time of standards, as well as molecular mass and structural information obtained from the MS detector during MS/MS experiments.

### 4.5. Identification of Unknown Compounds by LC-MS

The compounds in each sample were identified based on the retention times of standards, as well as molecular mass and structural information obtained from the MS detector during MS/MS experiments. Tandem mass spectrometric data were utilized to search for molecular structures using CSI:FingerID, a tool developed by Friedrich Schiller University, Jena, Germany. CSI:FingerID (linked to PubChem) is a web-based service that integrates isotope pattern analysis from MS spectra with fragmentation pattern analysis from MS/MS spectra, enabling the prediction of molecular fingerprints [49,50,51]. The CANOPUS algorithm was also included to predict compound classes from the molecular fingerprints generated by CSI:FingerID without involving any database search [52]. It is particularly useful for providing structural information when neither spectral nor structural reference data are available. CANOPUS outputs compound classifications based on the ClassyFire taxonomy [53]. ClassyFire provides a hierarchical chemical classification of small molecules. Simultaneously, tandem spectra from MoNA (MassBank of North America) were downloaded and used for compound annotation in MetaboScape 4.0.4 (Bruker Daltonik, Germany) by comparing the assigned MS/MS spectra with the MoNA spectral database. The results table (Supplementary Material) includes the compound class (computed using CANOPUS) and/or the predicted compound name (determined using MoNA and/or CSI:FingerID). In some cases, duplicate records appeared in the results table, indicating that CSI:FingerID identified multiple similar structures with high scores. The MS/MS spectra for certain compounds were recorded in both positive- and negative-ion modes, and the highest scores were reported. The MoNA match score is presented as a value ranging from 0 to 1000, while the CSI:FingerID score is calculated as Tanimoto similarity, which represents the percentage similarity of the top-ranked predicted fingerprints among all compounds. The retention times of all compounds were provided; however, it is possible that some spectra recorded during MS/MS experiments were actually MS^3^ experiments due to in-source fragmentation. Such false-positive records were excluded when an aglycone and its glycoside were identified at the same retention time. Since compound concentrations were calculated based on available standards, it is possible to compare the content of a given compound between samples. However, the concentrations of different compounds should not be compared, even when computed using appropriate standards.

### 4.6. DPPH Analysis

A ground sample (10 g) was placed in an Erlenmeyer flask and incubated with 100 mL of distilled water for 24 hours at room temperature. The sample was then filtered and centrifuged at 3000 rpm for 10 min. The capacity of the sample extracts to scavenge the stable free radical 2,2-diphenyl-1-picrylhydrazyl (DPPH) was estimated according to the method of Sanchez-Moreno et al. [54]. DPPH (1 mM, 0.25 mL) was dissolved in methanol, and then, 0.025–0.15 mL of the water extract samples were added. Absorbance was measured at 517 nm after 30 minutes of incubation at room temperature in darkness. The results were expressed as mg Trolox per g of dry matter (d.m.) extract.

### 4.7. Ferrous Ion-Chelating Activity

The results were estimated using the method described by Tang et al. [55]. The assay involved the colorimetric measurements of the degree of color loss in iron (II) chloride (2 mM, 0.1 mL) complexes with ferrozine (5 mM, 0.2 mL) caused by the extracts, with values in the range of 0.5–2 mL. The wavelength of measurement was 562 nm. The results were expressed as mg EDTA/g of dry matter extract. 

### 4.8. Total Phenolic Compounds (TPC)

The Folin–Ciocalteu reagent (FCR) method was adopted to estimate the total amounts of phenolic substances in the sample extracts [56]. The volume of the sample (0.025–0.2 mL of water extract) was stirred with distilled water (final volumes of water extract with water were 10 mL) and 0.5 mL of the FCR. The investigated material was blended with 1 mL of saturated sodium carbonate solution. Then, the mixture was incubated for 30 minutes at room temperature, and the absorbance was measured at 750 nm (Jena, Germany, Analytic Jena, Specord 40). The results were presented in mg of gallic acid equivalent per gram of dry matter extract (mg of GAE/g of d.m. extract).

### 4.9. Physiological State of Plants

At the BBCH 73 phase, parameters linked to the photosynthesis and water status of the plants were taken on the youngest expanded leaf of ten plants per condition in four replications. Chlorophyll fluorescence parameters, the maximum photochemical efficiency of PSII (Fv/Fm), effective quantum yield of photosystem II (Yield), and electron transport rate (ETR), were measured using a portable modulated chlorophyll fluorometer (OS5p, Opti-Sciences, Inc., Hudson, NY, USA) with a PAR Clip that allowed for the measurement of PAR and leaf temperature, according to Sulewska et al.’s protocol settings [57]. The net photosynthetic rate (A), leaf transpiration rate (E), the leaf stomatal conductance (Gs), and the intercellular CO_2_ concentration (Ci) were quantified by using a portable photosynthesis system (LCpro-SD, ADC BioScientific Ltd., Hoddesdon, UK) with a narrow leaf chamber (area: 5.8 cm^2^). The water use efficiency (WUE) was calculated as WUE = A/E. Measurements were taken on plants with a visible loss of turgor in the leaves 14 days after the abandonment of watering when the soil humidity of the drought-treated pots was at the level <10% by volume. In order to trace the regeneration of plants, they were irrigated again in the same way as at the beginning of the experiment. The regeneration of plants after undergoing drought stress was assessed after 6 days of irrigation (24 days from the start of measurements).

### 4.10. Statistical Analysis

The results reported in this study represent the mean of a few replications. To ensure the objectivity of the inferences, the recorded results were subjected to statistical analysis. A one-way analysis of variance (ANOVA) was conducted to determine the significance of differences between means using Tukey’s test. Differences were considered statistically significant at a significance level of *p* < 0.05. Principal Component Analysis (PCA) was used to reduce the dimensionality of the data and represent the samples in a new coordinate system. All results were analyzed using Statistica software (v. 13.3, StatSoft, Tulsa, OK, USA).

## Figures and Tables

**Table 1:** Dry matter, protein, lipid content, and the composition of total, soluble, and insoluble dietary fiber composition in selected parts of buckwheat plant: leaves; stems; seeds; and husk (g/100 g of dry powdered product).

		DM	Protein	Lipids	IDF	SDF	TDF
Leaves	S	91.3 ^b^	9.78 ^c^	1.74 ^c^	47.98 ^d^	0.42 ^e^	48.4 ^d^
	N	91.18 ^b^	12.89 ^b^	2.44 ^ab^	48.71 ^d^	0.42 ^e^	49.13 ^d^
Stems	S	93.01 ^a^	2.86 ^f^	0.47 ^d^	73.19 ^a^	3.46 ^b^	76.65 ^a^
	N	93.43 ^a^	5.08 ^e^	0.57 ^d^	72.16 ^a^	4.04 ^a^	76.20 ^a^
Seeds	S	89.62 ^c^	11.89 ^b^	2.15 ^b^	15.27 ^e^	0.09 ^f^	15.36 ^e^
	N	88.91 ^c^	16.11 ^a^	2.58 ^a^	15.59 ^e^	0.03 ^f^	15.62 ^e^
Husk	S	91.05 ^b^	8.23 ^d^	2.22 ^ab^	61.21 ^b^	2.34 ^d^	63.55 ^b^
	N	90.84 ^b^	8.59 ^d^	2.23 ^ab^	52.16 ^c^	2.53 ^c^	54.69 ^c^

Explanation: S—stressed; N—non-stressed; DM—dry matter; IDF—insoluble dietary fiber; SDF—soluble dietary fiber; TDF—total dietary fiber; ^a–f^ different letters in columns mean a significant difference (*p* < 0.05) between two water treatments according to ANOVA Tukey test.

**Table 2:** DPPH scavenging activity, ferrous ion-chelating activity, and total content of phenolic compounds in selected parts of buckwheat plant: leaves; stems; seeds; and husk.

		DPPH * (mg/g d.m. of Extract)	Ferrous Ion-Chelating Activity ** (mg/g d.m. of Extract)	TPC *** (mg/g of d.m. Extract)	Total Phenolics (mg/100 g of d.m. Product)
Leaves	S	208.03 ^b^	3.51 ^fg^	296.18 ^a^	266.33 ^c^
	N	257.21 ^a^	2.17 ^g^	152.76 ^b^	309.32 ^a^
Stems	S	27.45 ^e^	10.49 ^e^	58.40 ^d^	113.56 ^g^
	N	28.31 ^e^	7.83 ^ef^	65.57 ^c^	126.64 ^f^
Seeds	S	22.69 ^f^	57.89 ^d^	28.94 ^f^	233.54 ^e^
	N	22.21 ^f^	81.22 ^c^	31.19 ^e^	246.24 ^d^
Husk	S	43.59 ^d^	210.48 ^b^	43.3 ^g^	247.96 ^d^
	N	53.21 ^c^	439.44 ^a^	43.65 ^g^	276.77 ^b^

^a–g^ different letters in columns mean a significant difference (*p* < 0.05) between two water treatments according to ANOVA test; * expressed as a Trolox; ** expressed as EDTA; *** expressed as a gallus acid.

**Table 3:** The effect of water stress on chlorophyll fluorescence parameters (non-nominated units) of *F. tataricum* fresh leaves.

Water Treatment	During Drought Stress		After Regeneration	
	Non-Stressed	Stressed	Non-Stressed	Stressed
^1^ Fv/Fm	0.841 ^a^	0.823 ^b^	0.746 ^a^	0.775 ^a^
ETR	13.67 ^b^	43.40 ^a^	12.20 ^a^	10.64 ^a^
Yield	0.27 ^a^	0.30 ^a^	0.24 ^a^	0.21 ^a^

^1^ Fv/Fm—maximum quantum efficiency of PSII photochemistry; ETR—electron transport rate; Yield—effective quantum yield of photosystem II. ^a,b^ different letters in lines mean a significant difference (*p* < 0.05) between two water treatments according to ANOVA test.

## Data Availability

The data presented in this study are available on request from the corresponding author, as the larger-scale experiments are still ongoing.

## Supplementary Materials

The following supporting information can be downloaded at https://www.mdpi.com/article/10.3390/molecules30020270/s1, Table S1: Antioxidative compounds found in Tartary buckwheat leaves, stems, seeds, and husk (µg/g of product).
